# Perceived organizational Support and Nurses’ Caring Behaviors: The Serial Mediating Roles of Perceived Stress and Career Calling

**DOI:** 10.1155/jonm/8556856

**Published:** 2026-05-06

**Authors:** Wenfen Zhu, Qian Wu

**Affiliations:** ^1^ Department of Primary Nursing, School of Nursing, Chongqing Medical University, Chongqing, China, cqmu.edu.cn; ^2^ Department of Nursing, The First Affiliated Hospital of Chongqing Medical University, Chongqing, China, cqmu.edu.cn

**Keywords:** career calling, caring behaviors, clinical nurses, perceived organizational support, perceived stress, serial mediation

## Abstract

**Aim:**

To examine the association between perceived organizational support (POS) and nurses’ caring behaviors and to explore the serial mediating roles of perceived stress and career calling (CALL) in this relationship.

**Background:**

Caring behaviors are essential for high‐quality nursing care and patient satisfaction. However, heavy workloads and psychological pressure may weaken nurses’ caring performance. POS, as a key job resource, may be associated with caring behaviors alongside lower perceived stress and stronger intrinsic motivation, such as CALL.

**Methods:**

A cross‐sectional survey was conducted among 648 nurses from six tertiary hospitals in Chongqing, China. Standardized questionnaires were used to assess POS, perceived stress, CALL, and caring behaviors. The hypothesized serial mediation model was tested using structural equation modeling (SEM) with bias‐corrected bootstrapping, and additional PROCESS analysis was performed to verify robustness.

**Results:**

Organizational support was positively associated with caring behaviors (*β* = 0.33, *p* < 0.001), and a significant indirect association via perceived stress and CALL (*β* = 0.12, 95% CI [0.07, 0.24]). The model explained 26% of the variance in caring behaviors (*R* ^2^ = 0.26), and the indirect path accounted for about 26.7% of the total effect. Findings were consistent in PROCESS analyses and were in line with the hypothesized serial mediation model.

**Conclusion:**

POS was associated with nurses’ caring behaviors, both directly and through an indirect pathway involving perceived stress and CALL. These findings suggest that perceived stress and CALL may represent potential pathways linking organizational support with caring behaviors.

**Implications for nursing management:**

Nurse managers may consider fostering supportive organizational environments, implementing stress management interventions, and developing programs that help strengthen nurses’ CALL, as these factors may be associated with more favorable caring behaviors and nursing practice.

## 1. Introduction

Nurses’ caring behaviors are central to the nursing profession, reflecting humanistic values, professional responsibility, and commitment to patient care. High levels of caring behaviors can enhance patient satisfaction, trust, recovery, care quality, and safety [1, 2] and have been associated with positive nursing outcomes, including greater psychological comfort and lower anxiety among patients [3, 4]. Consequently, exploring the factors affecting nurses’ caring behaviors has important theoretical and practical value.

Perceived organizational support (POS) is a key external resource influencing nurses’ caring behaviors. It refers to employees’ perceptions that the organization values their contributions and cares about their well‐being [5]. Higher levels of POS can alleviate nurses’ psychological burdens, enhance their sense of belonging and professional identity, and motivate positive work behaviors [6]. Nurses who perceive strong organizational support may be more likely to express empathy, communicate effectively, and devote more time to meeting patients’ needs [7]. Conversely, nurses lacking supportive work environments are more prone to frustration and apathy, exhibiting lower levels of caring behaviors [8]. Thus, organizational support may be an important contextual factor associated with positive work behaviors. However, external support alone is insufficient to fully explain nurses’ caring behaviors, and it is necessary to further explore the internal psychological pathways that may be associated with these behaviors.

These internal psychological mechanisms are closely related to the various challenges nurses encounter in clinical practice. With increasing work intensity, staffing shortages, and the growing complexity of patient needs, nurses commonly endure high levels of work pressure and psychological burden [9]. High‐pressure environments can undermine nurses’ emotional stability and empathy, leading to burnout and job alienation, which reduce willingness and frequency to engage in caring behaviors [7, 10]. Research indicates that work stress and emotional exhaustion are key psychological factors inhibiting nurses’ caring behaviors [9, 11].

According to the Job Demands–Resources (JD‐R) model, work outcomes (such as work engagement or caring behaviors) result from the dynamic equilibrium between job demands and resources [12]. High levels of organizational support, as a job resource, can reduce nurses’ psychological exhaustion, enhance positive emotions, and increase professional commitment [12, 13]. Perceived stress, as a manifestation of job demands, reflects the process of psychological resource depletion [14]. Studies have shown that organizational support is associated with nurses’ positive behaviors and better mental health by reducing perceived stress [15, 16]. Concurrently, the Conservation of Resources (COR) theory posits that stress arises when vital resources are threatened or depleted, while acquiring new resources aids in restoring psychological energy [14]. Within this framework, perceived stress embodies the process of resource depletion, while career calling (CALL) represents an intrinsic psychological resource that stimulates professional passion and a sense of purpose [17]. Existing research indicates that CALL is associated with lower stress and higher levels of work engagement and caring behaviors [18, 19]. Nurses with a strong sense of CALL typically derive greater meaning from their work and maintain positive professional attitudes under stress [19].

Although previous studies have separately examined the associations of organizational support, perceived stress, and CALL on nurses’ work outcomes, these lines of research remain insufficiently integrated. In particular, a unified theoretical framework is still lacking to explain how external organizational resources may be associated with caring behaviors through internal psychological processes. Moreover, CALL has frequently been examined as either a direct antecedent of positive work outcomes or a personal psychological resource that is linked to lower burnout and other stress‐related outcomes [17, 20, 21]. Guided by the JD‐R model and COR theory, this study conceptualizes CALL as a meaning‐based intrinsic resource that is more likely to be activated when perceived stress is lower. Specifically, POS is viewed as a critical job resource associated with lower perceived stress, which in turn is associated with stronger CALL and higher levels of nurses’ caring behaviors.

Although previous research has conceptualized CALL as an antecedent or buffering resource that mitigates stress [17, 20, 22], the proposed model in the present study places perceived stress before CALL based on the JD‐R and COR perspectives. Perceived stress reflects ongoing strain and resource depletion that may constrain meaning‐making and reduce the salience of CALL [12, 14]; conversely, lower stress may allow greater psychological capacity to re‐engage with work meaning and purpose, which may be associated with stronger CALL [14, 22]. Accordingly, a serial mediation model is proposed in which organizational support is hypothesized to be negatively associated with perceived stress, perceived stress is hypothesized to be negatively associated with CALL, and CALL is hypothesized to be positively associated with caring behaviors, capturing a sequential “stress reduction⟶meaning activation” pathway. This framing highlights the conceptual novelty of the study and may offer tentative implications for nursing administration.

**FIGURE 1:**
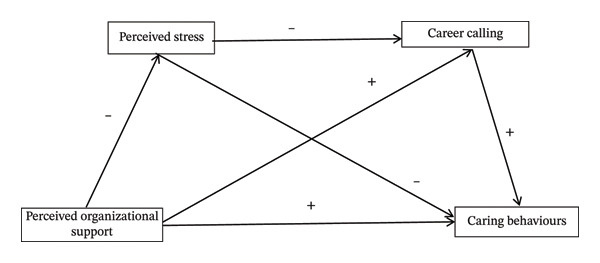
Hypothesized model.

Based on the above theoretical and empirical foundations, this study proposes the following hypotheses (see Figure 1).

**Hypothesis 1.**

POS is positively associated with nurses’ caring behaviors.

**Hypothesis 2.**

POS may be indirectly associated with nurses’ caring behaviors through perceived stress.

**Hypothesis 3.**

POS may be indirectly associated with nurses’ caring behaviors through CALL.

**Hypothesis 4.**

POS may be indirectly associated with caring behaviors through a serial indirect pathway involving lower perceived stress and higher CALL.

## 2. Methods

### 2.1. Design and Sample

**FIGURE 2:**
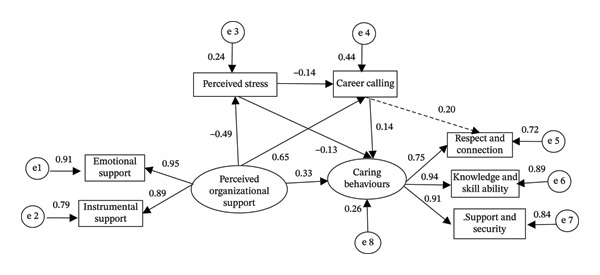
Mediation model with perceived stress and career calling as mediators. Note: Structural equation: caring behaviors = 0.33 × perceived organizational support−0.13 × perceived stress+0.14 × career calling, *R* ^2^ = 0.26; *R* ^2^ = 0.24 for perceived stress and *R* ^2^ = 0.44 for career calling.

This study employed a cross‐sectional survey design. Convenience sampling was used to recruit clinical nurses from six tertiary public hospitals located in the urban districts of Chongqing, China, each with more than 500 beds. Participants were recruited from general wards across the participating hospitals. Nurses working in highly specialized units such as emergency departments and intensive care units were not included in order to reduce heterogeneity in work environments. Inclusion criteria for participants were (i) being a licensed nurse legally registered in China (holding a valid Nurse Practice Certificate issued by the National Health Commission of China); (ii) possessing at least 2 years of clinical nursing experience (to reduce heterogeneity attributable to the novice transition period and ensure stable exposure to clinical demands); and (iii) being full‐time employees. Based on the basic sample size requirements for structural equation modeling (SEM) analysis [23], the recommended sample size ranged from 200 to 500, based on a recommended participant‐to‐free parameter ratio of 10:1 and 15:1. As shown in Figure 2, the final serial mediation model in this study contained 20 free parameters (12 weights and 8 variances). Therefore, the required sample size ranged from 200 to 300. In this study, a total of 648 clinical nurses were recruited.

### 2.2. Procedure

From September to October 2025, 10 trained research assistants, assisted by hospital nursing administrators, visited clinical departments to distribute questionnaires. They explained the purpose and procedures of the study to eligible participants and obtained signed informed consent. All questionnaires were completed online. On‐site research assistants provided only technical assistance (e.g., how to access the survey link) and did not observe responses or influence answers. Participants were assured of anonymity and confidentiality, and the design minimized response bias. The questionnaire included five self‐assessment sections: POS, perceived stress, CALL, caring behaviors, and demographics and took about 6–10 min to complete. Submissions were collected on‐site. Participation was entirely voluntary, and nurses could refuse or withdraw at any time without penalty. Nursing administrators assisted only with coordination and did not have access to identifiable data or individual responses. For participants who consented but had not yet completed the survey, the system sent nonpersonalized reminders during a 3‐day response window (maximum once every 12 h) to reduce missing data rather than to pressure participation. Invalid questionnaires were identified based on the following criteria: (i) Completion time under 3 min or exceeding 18 min; (ii) all answers were identical or followed a repetitive pattern (e.g., “1, 2, 3”); and (iii) any missing responses within the questionnaire. This study was approved by the Ethics Review Committee of Chongqing Medical University (approval no.: 2025–068).

### 2.3. Instruments

#### 2.3.1. Caring Behaviors

Caring behaviors were measured using the Caring Behavior Inventory‐24 (CBI‐24). Developed on the basis of Watson’s theory of human caring, this scale comprises three dimensions: Support and Security, Knowledge and Skill Ability, and Respect and Connection [1, 24]. Each dimension includes 8 items, totaling 24 items. The CBI‐24 was scored on a 6‐point Likert scale ranging from 1 (*never*) to 6 (*always*), with higher scores indicating stronger caring behaviors. The Chinese version, translated and validated by Da et al. has demonstrated good reliability and validity among Chinese nurses [25]. In the present study, Cronbach’s *α* coefficients for the three dimensions and the total scale were 0.951, 0.909, 0.955, and 0.973, respectively.

#### 2.3.2. Perceived Stress and Career Calling

The perceived stress of nurses was assessed with the Perceived Stress Scale (PSS). This scale was developed by Cohen et al. and later translated into Chinese by Yang Tingzhong in 2003 [26]. The scale consists of 14 items assessing individuals’ subjective perception of stress. Each item is rated on a 5‐point Likert scale ranging from 1 (*never*) to 5 (*always*), with total scores ranging from 14 to 70. Higher scores indicate higher perceived stress levels. The PSS has demonstrated good reliability and validity among Chinese populations. In this study, the Cronbach’s *α* coefficient of the total scale was 0.864.

CALL was measured using the Career Calling Scale developed by Dobrow et al. in 2011 [27]. The scale was adapted for the Chinese cultural context by Pei et al. [28] and later revised by Shen et al. [29], demonstrating high reliability and validity among Chinese nurses. The scale includes 12 items rated on a 5‐point Likert scale ranging from 1 (*strongly disagree*) to 5 (*strongly agree*), with higher total scores indicating a stronger sense of CALL. The Cronbach’s *α* coefficient of the scale was 0.955 in this study.

#### 2.3.3. POS

POS was measured using the Perceived Organizational Support Scale, which was developed by Chen based on the framework of Eisenberger’s research [30, 31] and later revised by Zuo to better reflect the characteristics of the nursing profession [32]. The scale contains two dimensions: emotional support and instrumental support, including 13 items in total. Each item was rated on a 5‐point Likert scale ranging from 1 (*strongly disagree*) to 5 (*strongly agree*), with higher total scores indicating higher levels of POS. The scale has shown good reliability and validity among Chinese nurses. In this study, the Cronbach’s *α* coefficients for emotional support, instrumental support, and the total scale were 0.972, 0.922, and 0.975, respectively.

#### 2.3.4. Sociodemographic Variables

Sociodemographic information included gender (female and male), age (years), education level(junior college or below, undergraduate, and postgraduate or above), working years, and marital status(single, married, and divorced).

### 2.4. Statistical Analysis

Descriptive statistics, correlation analysis, and reliability testing were conducted using SPSS 26.0. AMOS 24.0 was used to test the hypothesized serial indirect association involving perceived stress and CALL in the relationship between POS and caring behaviors. A two‐step SEM approach was adopted [33]. First, the measurement model was tested using confirmatory factor analysis (CFA). Second, the structural model was tested to evaluate the hypothesized relationships in this study. The overall model goodness of fit was assessed using the following criteria: a root mean square error of approximation (RMSEA) ≤ 0.06, *χ*2/df ≤ 5.00, a Tucker‒Lewis index (TLI) ≥ 0.95, a comparative fit index (CFI) ≥ 0.95, and a normed fit index (NFI) ≥ 0.90 [34, 35]. During model refinement, theoretically justified paths were added based on modification indices (MIs) and parameter changes [36]. The model was progressively refined according to MIs and covariance relationships, with iterative testing after each adjustment until satisfactory fit indices were obtained [34, 35, 37]. Direct and indirect path estimates were examined using bias‐corrected bootstrap sampling (5000 resamples) [38]. To confirm the robustness of the mediation results, an additional serial mediation analysis was also performed using the PROCESS macro (Model 6) in SPSS as a supporting analysis. In this analysis, demographic variables (gender, age, education level, working years, and marital status) were included as covariates to control for potential confounding effects. Because the primary purpose of the SEM analysis was to test the theoretical relationships among the main study variables, demographic variables were not included in the SEM model in order to maintain model parsimony.

## 3. Results

### 3.1. Demographic Characteristics of Participants

A total of 680 questionnaires were distributed. After review, 32 invalid responses were excluded. Among these, 4 were completed within 2 min, 6 exceeded 15 min, 12 showed identical or patterned answers across all items, and 10 contained missing data. Thus, 648 questionnaires with complete data were retained for analysis, yielding an effective recovery rate of 95.29% (648/680). The mean age of participants was 32.15 years (SD = 4.28). Participant demographic characteristics are shown in Table 1.

**TABLE 1:** Participant demographic information (*n* = 648).

Variables	*n* (%)	Variables	*n* (%)
Gender		Age	
Male	27 (4.2%)	< 30 years	179 (27.6%)
Female	621 (95.8%)	30–40 years	340 (52.5%)
		41–50 years	98 (15.1%)
		> 50 years	31 (4.8%)
Education level		Working years	
Junior college or below	88 (13.6%)	< 5 years	150 (23.1%)
Undergraduate	547 (84.4%)	5–10 years	294 (45.4%)
Postgraduate or above	13 (2.0%)	> 10 years	204 (31.5%)
Marital status			
Single	134 (20.7%)		
Married	487 (75.2%)		
Divorced	27 (4.2%)		

### 3.2. Descriptive Statistics and Variable Correlation Analysis

Table 2 presents the descriptive statistics and correlation analysis of the study variables. The results showed that POS was positively correlated with caring behaviors (*r* = 0.491, *p* < 0.01) and CALL (*r* = 0.629, *p* < 0.01). CALL also showed a positive correlation with caring behaviors (*r* = 0.466, *p* < 0.01). Perceived stress was negatively correlated with POS (*r* = −0.478, *p* < 0.01), caring behaviors (*r* = −0.361, *p* < 0.01), and CALL (*r* = −0.425, *p* < 0.01). In addition, Harman’s single‐factor test was conducted to assess common method bias. The first unrotated factor accounted for 36.3% of the total variance, which is below the commonly used 40% threshold, suggesting that common method bias was not a serious concern in this study.

**TABLE 2:** Mean scores and bivariate correlations between variables (*n* = 648).

	**Mean (SD)**	**Range**	**1**	**2**	**3**	**4**	**5**	**6**	**7**	**8**

1. Perceived stress	34.35 (8.271)	14–70	—							
2. Perceived organizational support	49.24 (10.506)	13–65	−0.478^∗∗^	—						
3. Emotional support	37.20 (8.439)	10–50	−0.474^∗∗^	0.993^∗∗^	—					
4. Instrumental support	12.04 (2.349)	3–15	−0.437^∗∗^	0.905^∗∗^	0.848^∗∗^	—				
5. Caring behaviors	125.76 (18.062)	24–144	−0.361^∗∗^	0.491^∗∗^	0.479^∗∗^	0.474^∗∗^	—			
6. Respect and connection	49.88 (8.848)	10–60	−0.369^∗∗^	0.503^∗∗^	0.496^∗∗^	0.468^∗∗^	0.939^∗∗^	—		
7. Knowledge and skill ability	26.99 (3.775)	5–30	−0.345^∗∗^	0.432^∗∗^	0.416^∗∗^	0.434^∗∗^	0.916^∗∗^	0.782^∗∗^	—	
8. Support and security	48.88 (6.781)	9–54	−0.288^∗∗^	0.412^∗∗^	0.398^∗∗^	0.411^∗∗^	0.928^∗∗^	0.761^∗∗^	0.863^∗∗^	—
9. Career calling	46.88 (8.594)	12–60	−0.425^∗∗^	0.629^∗∗^	0.626^∗∗^	0.567^∗∗^	0.466^∗∗^	0.504^∗∗^	0.385^∗∗^	0.369^∗∗^

^∗^ *p* < 0.05.

^∗∗^ *p* < 0.01.

### 3.3. Testing the Hypothesized Model and Parameter Estimates

#### 3.3.1. Measurement Model

Organizational support and caring behaviors were specified as first‐order latent constructs comprising two and three dimensions, respectively. The results of CFA revealed acceptable standardized factor loadings for the two organizational support dimensions (0.89–0.95). Factor loadings for the three dimensions of caring behaviors (0.84–0.94) were also satisfactory. Moreover, the squared multiple correlations (*R* ^2^) for all observed variables ranged from 0.71 to 0.91, indicating that the measurement model demonstrated good reliability and convergent validity.

#### 3.3.2. Structural Equation Model

The serial mediation effect was tested using SEM. The initial model showed poor fit indices (RMSEA = 0.108, *χ* ^2^/df = 8.56, NFI = 0.973, CFI = 0.976, and TLI = 0.949), prompting refinement. Model modifications were considered based on MIs and implemented only when they were theoretically plausible and did not alter the hypothesized core structure [34–36]. After examining the MI output, a single additional structural path from CALL to the “Respect and Connection” dimension of caring behaviors was added (MIs = 22.36 and par change = 6.33). This modification was theoretically justified because CALL reflects relationship‐oriented and altruistic values [18], while “Respect and Connection” represents the interpersonal and empathetic dimensions of caring behaviors [24, 39]. Importantly, this modification was added one at a time and the model was re‐estimated to confirm improvement in fit and the stability of the primary hypothesized paths.

After adding this path, the final model demonstrated good fit (*χ* ^2^/df = 2.83, CFI = 0.992, TLI = 0.984, and RMSEA = 0.053). The added path was statistically significant (*β* = 0.199, *p* < 0.001), and the direction and significance of all other main paths remained stable, as shown in Figure 2, which illustrates the relationships among organizational support, perceived stress, CALL, and caring behaviors. The standardized path coefficients indicated that organizational support was positively associated with caring behaviors (*β* = 0.33, *p* < 0.001) and was negatively associated with perceived stress (*β* = −0.49, *p* < 0.001), while it was positively associated with CALL (*β* = 0.58, *p* < 0.001). Perceived stress was also negatively associated with CALL (*β* = −0.14, *p* < 0.001) and caring behaviours (*β* = −0.13, *p* < 0.001), whereas CALL was positively associated with caring behaviours (*β* = 0.14, *p* < 0.001).

The model explained 26% of the variance (*R* ^2^ = 0.26) in caring behaviors, 24% (*R* ^2^ = 0.24) in perceived stress, and 44% (*R* ^2^ = 0.44) in CALL. Bootstrapping results showed a significant indirect association of organizational support on caring behaviors via perceived stress and CALL (*β* = 0.12 and 95% CI: [0.07, 0.24]), accounting for approximately 26.7% of the total effect, with the remaining 73.3% representing the direct effect (*β* = 0.33), as shown in Table 3.

**TABLE 3:** Total, direct, and indirect effects of perceived organizational support on caring behaviors in the structural equation model.

	**B**	**SE**	**95% CI (B)**	***β***	**95% CI (*β*)**	**Proportion**

Direct effect	1.06	0.241	(0.60.1.53)	0.33	(0.19.0.47)	73.3%
Indirect effect	0.47	0.141	(0.22.0.76)	0.12	(0.07.0.24)	26.7%
Total effect	1.53	0.148	(1.25.1.83)	0.45	(0.40.0.56)	100%

*Note: B* = unstandardized estimate; *β* = standardized estimate.

Abbreviations: CI = confidence interval; SE = standard error.

Goodness‐of‐fit indices: *χ* ^2^/df = 2.83, CFI = 0.992, TLI = 0.984, and RMSEA = 0.053. All path coefficients are standardized estimates, *p* < 0.01.

#### 3.3.3. PROCESS Findings

Additional PROCESS analyses further decomposed the indirect effects into specific mediation pathways. As shown in Table 4, perceived stress was involved in an indirect association between organizational support and caring behaviors (*β* = 0.061 and 95% CI: [0.025, 0.100]), while CALL also showed a significant independent indirect association (*β* = 0.130 and 95% CI: [0.073, 0.193]). Additionally, perceived stress and CALL jointly showed a significant serial indirect association (*β* = 0.017 and 95% CI: [0.007, 0.030]).

**TABLE 4:** Specific indirect effects of organizational support on caring behaviors in PROCESS.

Pathways	B	Boot SE (B)	95% CI (B)	β	Boot SE (*β*)	95% CI (*β*)
POS ⟶ PSS ⟶ CB	0.105	0.033	(0.042.0.172)	0.061	0.019	(0.025.0.100)
POS ⟶ CALL ⟶ CB	0.228	0.053	(0.125.0.330)	0.130	0.031	(0.073.0.193)
POS ⟶ PSS ⟶ CALL ⟶ CB	0.030	0.010	(0.013.0.052)	0.017	0.006	(0.007.0.030)

*Note:* PSS = perceived stress; CALL = career calling; *B* = unstandardized indirect effect estimate; *β* = standardized indirect effect estimate.

Abbreviations: CB = caring behaviors, CI = confidence interval, POS = perceived organizational support, SE = standard error.

Overall, these findings were consistent with the hypothesized serial indirect association linking POS to caring behaviors through perceived stress and CALL.

## 4. DISCUSSION

In high‐pressure clinical environments, nurses often face heavy workloads and psychological strain, which may hinder caring behaviors. Although previous studies have suggested an association between organizational support and caring behaviors [6, 7], a systematic explanation of how external resources interact with intrinsic motivation through internal psychological processes remains lacking. This study investigated the serial mediating roles of perceived stress and CALL in the association between POS and caring behaviors. It aimed to examine whether POS was associated with caring behaviors through perceived stress and CALL, in a pattern consistent with the “stress reduction—motivation activation” pathway.

### 4.1. POS and Caring Behaviors

This study found that POS was positively associated with nurses’ caring behaviors. In the SEM analysis, POS showed a significant direct association with caring behaviors (*β* = 0.33 and 95% CI: [0.19, 0.47]), indicating that nurses with higher POS were more likely to demonstrate caring behaviors in clinical practice. This aligns with the JD‐R model, which proposes that organizational resources can buffer work stress and motivate positive work behaviors [12]. Previous studies have also indicated that work environments with strong managerial support and sufficient resources are associated with higher levels of nurses’ caring behaviors [7, 40]. In addition, nurses with greater POS tend to report higher well‐being and stronger professional commitment, which may be related to more consistent and empathetic nursing practices [41]. These findings suggest that POS may represent an important external resource associated with caring behaviors. Accordingly, it may be useful for hospital administrators to consider optimizing the practice environment, strengthening resource allocation, and establishing effective feedback mechanisms, as these factors may be relevant to nurses’ capacity for compassionate care.

### 4.2. The Mediating Role of Perceived Stress

This study further found that perceived stress may partly account for the association between POS and nurses’ caring behaviors. POS was negatively associated with perceived stress (*β* = −0.49), indicating that supportive organizational environments may be linked to lower nurses’ psychological strain. In turn, perceived stress was negatively associated with caring behaviors (*β* = −0.13), suggesting that higher stress may be related to lower engagement in caring practices. Consistent with these pathways, the indirect effect of Perceived Organizational Support ⟶ Perceived Stress ⟶ Caring Behaviors was statistically significant (*β* = 0.061 and 95% CI: [0.025, 0.100]), suggesting an indirect association involving perceived stress. This finding is consistent with previous studies showing that organizational support has been associated with work stress and emotional exhaustion [42, 43]. Lower perceived stress may be related to better emotional stability and preserved psychological resources, which may in turn be associated with more favorable caring behaviors [42, 43]. From a clinical management perspective, these findings highlight the importance of stress‐reduction strategies within healthcare organizations. Nursing managers may consider implementing supportive workplace policies and stress management programs to help nurses cope with job demands and maintain positive emotional states, which may be relevant to patient‐centered care.

### 4.3. The Mediating Role of CALL

Another important finding of this study was that CALL showed the strongest indirect association between POS and caring behaviors. The indirect pathway Perceived Organizational Support ⟶ Career Calling ⟶ Caring Behaviors showed the largest indirect estimate (*β* = 0.130 and 95% CI: [0.073, 0.193]), indicating that CALL appeared to be an important correlate in the association between organizational support and caring behaviors. Nurses with greater POS tend to experience a stronger CALL, which, in turn, may be associated with higher levels of caring behaviors. Existing evidence suggests that POS is positively associated with nurses’ CALL, while CALL itself has been associated with stronger professional meaning, lower burnout, and better behavioral performance [17, 20, 44].

These results suggest that organizational support may be associated not only with external support conditions but also with nurses’ intrinsic motivation, including CALL. In practice, nursing managers may consider developing programs that support nurses’ professional identity and sense of mission, such as mentorship initiatives, reflective practice sessions, and professional value–based education. However, these strategies were derived from the observed associations and prior literature and were not directly evaluated in the present study.

### 4.4. The Serial Mediating Role of Perceived Stress and CALL

A more notable finding of this study was that POS was indirectly associated with nurses’ caring behaviors through the serial indirect association involving.

Perceived stress and CALL: Comparing the indirect pathways, the CALL pathway (Perceived Organizational Support ⟶ Career Calling ⟶ Caring Behaviors, *β* = 0.130) showed the largest indirect effect, followed by the perceived stress pathway (Perceived Organizational Support ⟶ Perceived Stress ⟶ Caring Behaviors, *β* = 0.061), whereas the serial pathway (Perceived Organizational Support ⟶ Perceived Stress ⟶ Career Calling ⟶ Caring Behaviors, *β* = 0.017) was the smallest. Despite its smaller magnitude, the serial pathway remained statistically significant, suggesting a sequential association linking perceived organizational support to caring behaviors through lower perceived stress and stronger CALL. Overall, this pattern is consistent with a “stress reduction ⟶ motivation activation” pathway, in which CALL appeared to be the more prominent correlate linking POS to nurses’ caring behaviors. This finding aligns with the core assumptions of the JD‐R model and the COR theory [12, 14]. According to the JD‐R model, organizational support, as a critical job resource, can buffer psychological stress arising from high job demands and be associated with stronger intrinsic motivational resources such as CALL, thereby facilitating positive work outcomes. Consistent with previous research, POS has been associated with lower nurses’ work stress and stronger professional commitment [6, 45], while lower stress levels are associated with higher caring behaviors [9]. Importantly, these findings suggest that CALL may not function merely as a stable personal attribute but rather as a meaning‐based motivational resource that becomes more salient when perceived stress is lower. In other words, the results are consistent with a sequential pathway in which lower perceived stress is associated with stronger CALL, which in turn is associated with higher caring behaviors. This dynamic perspective extends prior research by highlighting CALL as a mobilized resource within a resource‐related association rather than an isolated predictor. This is consistent with Dik and Duffy’s (2009) concept of “meaning‐driven motivation” and aligns with empirical evidence showing that CALL has been associated with work commitment and caring behaviors [20–22, 44].

Additionally, guided by the MIs and theoretical rationale [17, 19], this study incorporated a path between CALL and the “Respect and Connection” dimension in the final model. This adjustment significantly improved model fit and revealed the specific role of CALL within the caring behavior construct. This finding suggests that CALL primarily manifests through relational and empathic caring behaviors rather than technical or procedural aspects. This result aligns with the view that CALL embodies altruistic and relationship‐oriented values [17, 19] and may add to current understanding of how intrinsic motivation is statistically related to interpersonal expressions of care. Nevertheless, as this path was added during model refinement, its robustness should be further verified in diverse samples.

In summary, organizational support was directly associated with clinical nurses’ caring behaviors and was also indirectly associated with them through perceived stress and CALL. This serial pattern suggests a theoretically plausible explanation for how nurses’ caring behaviors may be associated with organizational and psychological factors. From a practical perspective, nurse managers may consider creating supportive work environments and exploring ways to support nurses’ CALL. These strategies may be relevant to nurses’ caring capacity and professional commitment under high‐pressure conditions. However, these recommendations should be interpreted cautiously because the present study did not test interventions directly, and further research is needed to confirm their effectiveness.

### 4.5. Implications for Nursing Management

The findings offer practical implications for nursing management. First, POS, as a key external resource, was positively associated with caring behaviors. Nurse managers may, therefore, consider ways to strengthen nurses’ perceptions of organizational support. Possible approaches informed by prior literature include fair workload distribution, open communication, and recognition of contributions, [46]. Second, perceived stress appeared to be an important variable linking organizational support and CALL, suggesting that reducing workplace stress may be relevant to caring behaviors. Hospitals may consider exploring approaches such as mindfulness‐based stress reduction, resilience training, and peer support, which have been reported in previous research to be associated with lower stress and burnout among nurses [47]. However, these approaches were not directly tested in the present study. Third, CALL appeared to be an important correlate in the observed association, suggesting that support for nurses’ CALL may also be relevant. Previous research has discussed mission‐oriented education, mentorship, and reflection as possible ways to support CALL [48, 49]. Overall, a comprehensive management approach that combines external resource provision (POS) with internal motivation enhancement (CALL) may be a useful direction for nursing management. Such integrated strategies may be relevant to nurses’ caring capacity and professional commitment, particularly in high‐demand and resource‐constrained healthcare settings.

### 4.6. Limitations

Several limitations should be acknowledged in this study. First, the use of a cross‐sectional survey limits the ability to infer directional associations among organizational support, perceived stress, CALL, and caring behaviors. Future research could apply longitudinal tracking or controlled experimental methods to clarify these potential causal links. Second, all measures relied on nurses’ self‐report, which might have been influenced by subjective perception or social desirability bias. Collecting information from multiple sources (such as supervisors, peers, or patients) would help increase the reliability of the results. Third, participants were recruited only from tertiary hospitals in Chongqing, meaning that local cultural and institutional contexts may restrict how far these findings extend to other regions. Subsequent studies should, therefore, involve diverse provinces and healthcare levels to strengthen external validity. In addition, this study focused primarily on the core theoretical variables specified in the model and did not include certain occupational characteristics, such as job title grade or administrative roles. These factors may also influence nurses’ work experiences and caring behaviors. Future studies could incorporate such career‐related variables to provide a more comprehensive understanding of the nursing work context. Finally, the additional path between CALL and the “Respect and Connection” dimension was added during model refinement, which, although theoretically justified, requires further confirmation in independent samples to ensure the robustness of the model.

## 5. Conclusions

This study suggests that POS was positively associated with nurses’ caring behaviors, both directly and indirectly, through perceived stress and CALL. These findings indicate that perceived stress and CALL may be relevant factors linking POS with caring behaviors. Strengthening organizational support systems and attending to nurses’ CALL may, therefore, be worthwhile considerations in relation to high‐quality nursing practice although these implications should be interpreted cautiously given the cross‐sectional design.

## Funding

No funding was received for this manuscript.

## Ethics Statement

This study was approved by the institutional review board of Chongqing Medical University Ethics Committee (approval no: 2025–068).

## Conflicts of Interest

The authors declare no conflicts of interest.

## Data Availability

The data that support the findings of this study are available from the corresponding author upon reasonable request.
